# Supplementary material for: Content-rich biological network constructed by mining PubMed abstracts
Source: BMC Bioinformatics. 2004 Oct 8;5:147. doi: 10.1186/1471-2105-5-147 (PMC528731; doi:10.1186/1471-2105-5-147)
Supplement: Additional File 2 — The original results of the above study (non-essential files are deleted to keep the file size under the limit set by BMC bioinformatics). [file 1471-2105-5-147-S2.bz2 › chilibotAdditionalFile2/dip20/24ID9219684E84/html/RASA1_HRAS.html]

 


 **RASA1** and **HRAS** 
  
Found 13 abstracts in PubMed, retrieved 13.  
 

 What does Google say? 
 PDF only 
| .edu only 

---

**Interactive relationship** (e.g. stimulation, inhibition, etc)

**Neutral relationship**- We therefore analyzed the interaction between the c rasH  [ **HRAS** ]  S39C protein and the potential target molecules GTPase activating protein  [ **RASA1** ]  GAP  [ **RASA1** ]  and the GAP  [ **RASA1** ]  related domain of NF 1, on the basis of stimulation of the mutant p21ras GTPase activity by these molecules in vitro.  Ref: 2038322 Mol Cell Biol, 1991

**Non-interactive relationship** (e.g. studied together, co-existance, homology, etc.)

- Platelet derived growth factor stimulation of GTPase activating protein  [ **RASA1** ]  tyrosine phosphorylation in control and c H ras  [ **HRAS** ]  expressing NIH 3T3 cells correlates with p21ras activation.  Ref: 1508192 Mol Cell Biol, 1992
- Preferential inhibition of the oncogenic form of RasH  [ **HRAS** ]  by mutations in the GAP  [ **RASA1** ]  binding effector domain.  Ref: 1899358 Cell, 1991
- We have sought activating substitutions in c H ras  [ **HRAS** ]  in the region encoding the effector domain, on the rationale that such mutations would dissociate effector function from negative regulation by GAP  [ **RASA1** ] .  Ref: 8246952 Mol Cell Biol, 1993
